# Microalgae in health care and functional foods: β-glucan applications, innovations in drug delivery and synthetic biology

**DOI:** 10.3389/fphar.2025.1557298

**Published:** 2025-03-04

**Authors:** Chao Li, Ming Du, Yujie Han, Wentao Sun, Zixi Chen, Qiong Liu, Hui Zhu, Liqing Zhao, Shuangfei Li, Jiangxin Wang

**Affiliations:** ^1^ College of Physics and Optoelectronic Engineering, Shenzhen University, Shenzhen, China; ^2^ School of Life Sciences and Food Engineering, Hanshan Normal University, Chaozhou, Guangdong, China; ^3^ Shenzhen Key Laboratory of Marine Bioresource and Eco-environmental Science, Shenzhen Engineering Laboratory for Marine Algal Biotechnology, Guangdong Provincial Key Laboratory for Plant Epigenetics, College of Life Sciences and Oceanography, Shenzhen University, Shenzhen, China; ^4^ College of Chemistry and Environmental Engineering, Shenzhen University, Shenzhen, China

**Keywords:** microalgae, health care, functional foods, β-glucan, drug delivery, synthetic biology

## Abstract

Microalgae are emerging as a key player in healthcare, functional foods, and sustainable biotech due to their capacity to produce bioactive compounds like β-glucans, omega-3 fatty acids, and antioxidants in an eco-friendly manner. This review comprehensively discusses the role of microalgae in healthcare and functional foods, focusing particularly on β-glucan therapeutics, drug delivery innovations, and synthetic biology applications. In healthcare, microalgae-derived compounds show immense promise for treating diseases, boosting immunity, and tackling oxidative stress. *Euglena*-derived paramylon, a type of β-glucan, has shown potential in various medical applications, including immunomodulation and anticancer therapy. Synthetic biology and bioprocess engineering are enhancing microalgae’s therapeutic and nutritional value, with applications in drug delivery and personalized medicine. To maximize the potential of microalgae, further research and development are needed to address scalability, regulatory alignment, and consumer acceptance, with a focus on interdisciplinary collaboration and sustainable practices to align healthcare innovation with environmental conservation.

## 1 Introduction

Microalgae represent an extraordinary resource in the realm of biotechnology, offering diverse applications in health, such as functional foods and nutraceuticals; nutrition, including protein-rich feedstocks; and environmental sustainability through carbon capture and wastewater treatment. Their unique capacity for photosynthesis and production of high-value bioactive compounds, including β-glucans, pigments, and pharmaceuticals, underpins their increasing importance in modern science and industry (Nethravathy et al., 2019; Ampofo and Abbey, 2022). The therapeutic effects of microalgae bioactive compounds and their applications in biomedical research have garnered growing interest (Huang et al., 2024). Studies of bioactive compounds from microalgae were provided in Table 1.

**TABLE 1:** Bioactive components from microalgae.

Species	Bioactive compound	Content	Clinical dose	Biological activity	References
*Spirulina* (*Arthrospira platensis*)	Phycocyanin	14% of dry weigh	2–8 g/day	Antioxidant, anti-inflammatory, neuroprotective	Romay et al. (2003)
*Chlorella vulgaris*	*Chlorella* growth factor (CGF)	10%–20% of dry weigh	4–10 g/day	Immune system support, detoxification, promotes cell repair	Merchant and Andre (2001)
*Haematococcus pluvialis*	Astaxanthin	1.5%–3% of dry weigh	4–12 mg/day	Antioxidant, UV protection, anti-aging, supports cardiovascular health	Ambati et al. (2014), Huang et al. (2024)
*Dunaliella* spp.	Beta-carotene	Up to 14% of dry weigh	2–6 mg/day	Antioxidant, precursor to vitamin A, supports eye health	Ben-Amotz and Levy (1996)
	Polysaccharides	5%–11% of dry weight	200–500 mg/day	Antioxidant, anti-inflammatory, promotes gut health	Hyrslova et al. (2022), Raposo et al. (2013)
*Nannochloropsis* spp.	Eicosapentaenoic Acid (EPA)	3%–5% of total fatty acids	250–500 mg/day	Anti-inflammatory, supports cardiovascular and brain health	Khozin-Goldberg et al. (2011)
	Sterols	0.5%–2% of dry weight	100–300 mg/day	Antioxidant, supports skin health, anti-inflammatory	Kim and Van (2012), Uthaiah et al. (2025)
*Porphyridium cruentum*	Polysaccharides	10%–15% of dry weight	100–300 mg/day	Antiviral, immune modulation, anti-inflammatory	Guzmán et al. (2001), Huang et al. (2024)
*Isochrysis galbana*	Fucoxanthin	0.1%–1% of dry weight	2–8 mg/day	Antioxidant, anti-obesity, supports metabolic health	Peng et al. (2011)
*Schizochytrium* spp.	Docosahexaenoic Acid (DHA)	30%–50% of total fatty acids	200–500 mg/day	Brain development, cognitive function, anti-inflammatory	Barclay et al. (1994)
*Tetraselmis* spp.	Lutein	0.5%–1.5% of dry weight	10–20 mg/day	Eye health, antioxidant, reduces risk of macular degeneration	Sánchez et al. (2008)
*Odontella aurita*	Omega-3 Fatty Acids	20%–30% of total fatty acids	250–500 mg/day	Cardiovascular health, anti-inflammatory, supports brain function	Tonon et al. (2002)
*Cylindrotheca fusiformis*	Fucoxanthin	0.1%–2.4% of dry weight	2–8 mg/day	Antioxidant, anti-obesity, anti-inflammatory, anticancer	Peng et al. (2011), Wang et al. (2018), Huang et al. (2024)
	EPA	25% of total fatty acids	250–500 mg/day	Cardiovascular health, anti-inflammatory	
*Euglena* spp.	β-Glucan (Paramylon)	70%–80% of dry weight	500–1,000 mg/day	Enhances immune response, antitumor, antioxidant, hypoglycemic effects, and other effects	Watanabe et al. (2013), Kim et al. (2019), Wu, et al., 2021; Lei et al. (2022), Li et al. (2024)

Among these, β-glucans—natural polysaccharides known for their immunomodulatory and antioxidant properties—have garnered significant attention for their potential in functional foods, nutraceuticals, and pharmaceuticals, such as their incorporation in paramylon-enriched supplements for immune enhancement and gut health (Murphy et al., 2020; Taylor et al., 2020). *Euglena*-derived paramylon, a β-(1→3)-glucan, is particularly notable for its high purity, ease of extraction, and versatile applications (Gissibl et al., 2019).

**FIGURE 1:**
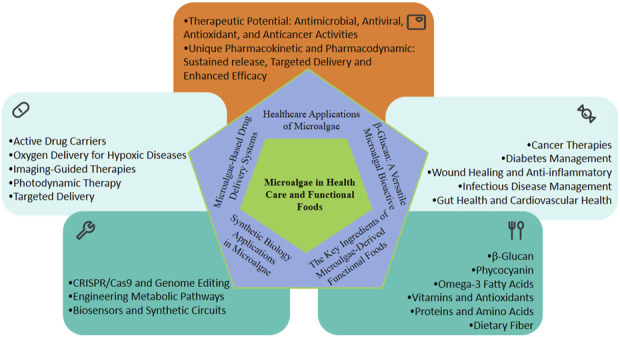
Microalgae in healthcare and functional food applications.

Another emerging frontier is the utilization of microalgae in drug delivery systems. Microalgal cells and their derivatives, such as lipids and biopolymers, have shown promise as carriers for therapeutic agents, leveraging their biocompatibility, bioavailability, and ability to target specific tissues (Vieira et al., 2020; Huang et al., 2024). These attributes, coupled with advancements in synthetic biology, position microalgae as pivotal players in addressing global challenges in health, energy, and environmental sustainability (Fernández et al., 2021; Naduthodi et al., 2021). Microalgae in healthcare and functional food applications were summarized in Figure 1.

Synthetic biology further expands the potential of microalgae by enabling precise genetic modifications to enhance productivity and introduce novel functionalities (Naduthodi et al., 2021). For example, CRISPR-Cas9 has been used to boost lipid biosynthesis in *Nannochloropsis oceanica*, while TALENs have optimized pigment production pathways in *Chlamydomonas reinhardtii* (Liu et al., 2024; Shin et al., 2024). These modifications have resulted in higher yields and more efficient bioproduct generation. Advances in genome editing tools, such as CRISPR-Cas9 and TALENs, have catalyzed the engineering of microalgae for sustainable production of biofuels, pigments, and therapeutic proteins (Fayyaz et al., 2020; Naduthodi et al., 2021).

This review provides a comprehensive analysis of the advancements in β-glucan research, drug delivery systems and synthetic biology applications, involving microalgae. These three areas were selected because they exemplify distinct yet interconnected facets of microalgae’s potential in biotechnology. β-Glucan research highlights the foundational bioactive properties of microalgae, synthetic biology showcases the tools and strategies to enhance these properties, and drug delivery systems demonstrate their practical applications in medicine. Together, they form a cohesive narrative that bridges natural bioactivity, engineered innovation, and real-world therapeutic impact, offering a holistic view of microalgae’s promise in health and sustainability.

## 2 β-Glucan from microalgae and applications

β-Glucans are natural polysaccharides composed of glucose monomers linked predominantly by β-(1→3), β-(1→4) and occasionally β-(1→6) glycosidic bonds. These compounds are widely distributed in nature, being found in cereals, fungi, yeast, and microalgae (Chiozzi et al., 2021; van Steenwijk et al., 2021; Lazaridou et al., 2007; Kumar et al., 2025). They have attracted significant scientific and commercial interest due to their broad range of biological activities, including immunomodulation, antioxidant effects, and potential applications in functional foods and pharmaceuticals. Among various sources, microalgae-derived β-glucans are particularly noteworthy for their unique structural properties, cost-effective production, and scalability (Murphy et al., 2023; Kumar et al., 2025). Studies of β-Glucan from microalgae were provided in Table 2.

**TABLE 2:** β-Glucan from microalgae.

Species	Construction	Content	Biological activity	References
*Chlorella pyrenoidosa*	Cyclic β-(1-2)-glucan	<5%	Bacterial virulence, symbiosis, and host-pathogen interactions	Reyes Suárez, et al. (2008)
*Euglena* spp.	Linear β-(1-3)-glucan	70%–80%	Immunological function enhancement Body defences strengthening Immunological reactions triggering Immunological function augmentation Prebiotics	Wu, et al. (2021)
*Pavlova mesolychnon*	Linear β-(1-3)-glucan	-		Myklestad and Granum, (2009)
*Sargassum henslowianum*	Linear β-(1-3)-glucan	-		Cui, et al. (2021)
*Ulva lactuca*	Linear β-(1-3, 1-4)-glucan	-	Cholesterol level reduction Diabetes management Structural functionality	Salmeán, et al. (2017)
*Kappaphycus alvarezii*	Linear β-(1-3, 1-4)-glucan	-		Salmeán, et al. (2017)
*Monodus subterraneus*	Linear β-(1-3, 1-4)-glucan	18.8%		Frick, et al. (2023)
*Micrasterias* spp.	Linear β-(1-3, 1-4)-glucan	-		Salmeán, et al. (2017)
*Isochrysis galbana*	Branched β-(1-3, 1-6)-glucan	<10%	Immunomodulators Anticancer activity Antioxidant characteristics	Sadovskaya, et al. (2014)
*Chaetoceros debilis*	Branched β-(1-3, 1-6)-glucan	<10%		Størseth, et al. (2006)
*Laminaria* spp.	Linear β-(1-3)-glucan	1%–32%		Kadam, et al. (2015)
*Nannochloropsis gaditana*	Branched β-(1-3, 1-6)-glucan	-		Vogler, et al. (2018)
*Scenedesmus ovalternus*	Branched β-(1-3/1-6)-glucan	30%–45%		Schulze, et al. (2016)
*Scenedesmus obtusiusculus*	Branched β-(1-3/1-6)-glucan	7.8%		Schulze, et al. (2016)

### 2.1 Unique advantages of *euglena*-derived β-glucan (paramylon)

*Euglena gracilis* is a unique microalga that produces a highly linear form of β-(1→3)-glucan called paramylon. This distinguishes it from other sources of β-glucans due to several specific advantages. First, *Euglena* biomass contains an exceptionally high proportion of paramylon, ranging from 70% to 80% of its dry weight, which is substantially higher than β-glucan yields from yeast (30%–50%) or cereals (5%–8%) (Wu et al., 2021; Lazaridou et al., 2007; Chiozzi et al., 2021). The high content of paramylon significantly enhances its economic viability for industrial applications.

Unlike other sources, where β-glucans are embedded in the cell wall and require enzymatic or chemical disruption for extraction, paramylon is stored as intracellular granules. This simplifies its extraction process, reducing processing costs and preserving the molecule’s structural integrity (Guo et al., 2020; Chiba et al., 2024). Additionally, paramylon’s linear β-(1→3) structure is believed to enhance its bioactivity, making it highly effective in modulating immune responses and exhibiting antioxidant properties (Barsanti and Gualtieri, 2019; Gao et al., 2022). *Euglena* β-glucan (paramylon) studies were provided in Table 3.

**TABLE 3:** *Euglena* β-glucan (paramylon) studies in animals and aquaculture.

Bioactivities	Mechanism	Study subject	Study design	References
Immunity	Activation of immune cells	Mice, Human (*In vitro*), Fish	Co-culture (0.01–10 mg/mL, 30 min-24 h), oral administration (2% w/w diet, 50 days)	Russo et al. (2017), Suzuki et al. (2018), Phillips et al. (2019), Guo et al. (2020), Wu et al. (2023a)
	Regulation of immune pathways	Mice	Oral or injection administration (250–400 mg/kg body weight, 2 h-50 days)	Suzuki et al. (2018), Yasuda et al. (2020), Ishibashi et al. (2022), Yang et al. (2022), Chiba et al. (2024), Jo et al. (2024)
	Immune-enhancing effects	Fish, Pigs	Feeding administration (0–800 mg/kg diet, 17–84 days)	Skov et al. (2012), Yamamoto et al. (2018), Kim et al. (2019)
	Prevention of allergic diseases	Mice	Oral administration (0.1–0.9 g/kg body weight, 46 days)	Sugiyama et al. (2010)
	Alleviation of immunosuppression	Mice	Oral administration (250 mg/kg body weight, 19 days)	Yang et al. (2022)
Antiviral	Anti-HIV	Human (*In vitro*)	Co-culture (0.06–1,000 μg/mL, 5 days)	Koizumi et al. (1993)
Antioxidant and free radical scavenging	Direct scavenging	*In vitro*	Co-culture (0.08–10 mg/mL, 24 h)	Phillips et al. (2019), Lei et al. (2022)
	Damage protection	Mice	Oral administration (2 mg/kg body weight, 11 weeks), feeding administration (0.3 g/100 g diet, 8 weeks)	Watanabe et al. (2013), Okouchi et al. (2019)
Anti-inflammatory	Cytokine inhibition	Rat	Oral administration (5% w/w diet, 8 weeks)	Nagayama et al. (2020)
	Cytokine enhancement	*In vitro*	Co-culture (0.08–10 mg/mL, 24 h)	Phillips et al. (2019)
Antitumor and cancer support	Oxidative stress reduction	Mice	Oral administration (2 mg/kg body weight, 11 weeks)	Watanabe et al. (2013)
	Tumor apoptosis	Mice	Oral administration (2 mg/kg body weight, 11 weeks)	Watanabe et al. (2013)
Antibacterial	Chemical modification	*Escherichia coli* and *Staphylococcus aureus*	Co-culture (30 mg/mL, 24 h)	Gao et al. (2022)
Activate epidermal cells	Inducing the production of secretory factors from intestinal cells	Human (*In vitro*)	Co-culture (100 μg/mL, 15 days)	Ieiri et al. (2021)
Diabetes metabolism and health	Insulin sensitivity	Mice	Gavage administration (400 mg/kg/d, 4 weeks)	Li et al. (2024)
	Hypoglycemic effects	Mice, Rat	Oral administration (2% w/w diet, 10 weeks), gavage administration (400 mg/kg/d, 4 weeks)	Shimada et al. (2016), Li et al. (2024)
	Bile acid sequestration	*In vitro*	Oral administration (2% w/w diet, 5 weeks)	Shibakami et al. (2018)
Liver protection	Anti-inflammatory effects	Mice	Oral administration (0.1–0.9 g/kg body weight, 46 days), gavage (400 mg/kg body weight, 2–24 h)	Sugiyama et al. (2010), Xie et al. (2021)
	Lipid metabolism regulation	Mice	Feeding administration (51.2 g/kg body weight, 12 weeks)	Aoe et al. (2021)
	Anti-fibrotic activity	Mice	Oral administration (3 g/day, 27 days)	Nakashima et al. (2019)
Gut regulation and microbiome regulation	Prebiotic effects	Human Mice	Oral administration (1.8–2 g/day, 14–30 days)	Nakashima et al. (2021), Song et al. (2022)
	Gut motility enhancement	Mice	Oral administration (1.8 g/kg/day, 2 weeks)	Song et al. (2022)
	Gut dysbiosis protection	Mice	Oral administration (10 mg/kg body weight, 45 days)	Taylor et al. (2020)
Kidney protection	Fibrosis inhibition and renal function improvement	Rat	Oral administration (5% w/w diet, 8 weeks)	Nagayama et al. (2020)
Wound healing and tissue repair	Promotion of angiogenesis (HIF-1α-VEGF pathway)	Mice	Wound dressing (Paramylon hydrogel, 1–9 weeks)	Lei et al. (2022)
	Reduces inflammation in skin wounds while promoting collagen deposition and cell proliferation	Mice	Wound dressing (Paramylon hydrogel, 1 day-9 weeks)	Li et al. (2021), Lei et al. (2022), Lei et al. (2024)
	Physical barrier and tissue support	Mice	Wound dressing (Paramylon film, 5 days)	Yasuda et al. (2018)
Weight management and anti-obesity	Reduction of visceral fat and improvement of obesity markers	Humans	Oral administration (2.6 g per day, 12 weeks)	Aoe et al. (2023)
	Regulation of lipid metabolism	Mice	Oral administration (51.2 mg/kg body weight, 12 weeks)	Aoe et al. (2021)
	Synergistically modulation of gut microbiota	Mice	Feeding administration (0.3 g/100 g diet, 8 weeks)	Okouchi et al. (2019)
Plant regulation	Enhanced water-use efficiency, improved photosynthesis rate, and significant alteration in xylem cytokinin and abscisic acid levels	*Solanum lycopersicum* L	Mix with nutrient solution (90–500 mg/L, 96 h)	Scartazza et al. (2017)
Modulation of peripheral circadian clocks	Modulating clock gene expression	Mice	Oral administration (10 mg/kg body weight, 3 days)	Ryan et al. (2023)

### 2.2 Comparative analysis with other β-glucan sources

*Euglena*’s paramylon outperforms other β-glucan sources in several critical aspects, including yield, ease of extraction, and bioactivity. For instance, cereal-derived β-glucans, such as those from oats and barley, are primarily branched (β-(1→3, 1→4)) and require extensive processing that can degrade their structure and reduce bioactivity (Henrion et al., 2019; Lante et al., 2023). Similarly, yeast-derived β-glucans often contain allergenic impurities and are less effective as immunomodulators compared to the linear β-(1→3) structure of paramylon (Yamamoto et al., 2020; Kumar et al., 2025).

In terms of industrial applications, *Euglena*-derived β-glucan excels in functional food formulations due to its high insolubility and stability under various conditions. Its high purity and bioactivity also make it a promising candidate for pharmaceutical applications, such as immunotherapies and antioxidant treatments (Nakashima et al., 2017; Yamamoto et al., 2018; Yasuda et al., 2020; Huang et al., 2023).

### 2.3 Biological activities and applications of paramylon

Paramylon demonstrates a wide range of biological activities, supported by both *in vitro* and *in vivo* studies. It has been shown to enhance immune responses by activating macrophages, dendritic cells, and natural killer cells. For example, studies in mice and human cell cultures have reported increased cytokine production, including TNF-α and IL-6, following paramylon administration (Nakashima et al., 2017; Russo et al., 2017). Immune activation of murine RAW264.7 macrophages by sonicated and alkalized paramylon from *Euglena* was also reported (Guo et al., 2020). Additionally, paramylon’s prebiotic effects have been demonstrated in probiotics (Dai et al., 2022) and animal models, where it modulates gut microbiota and improves gut health (Taylor et al., 2020).

Paramylon also exhibits potent antioxidant properties, effectively scavenging reactive oxygen species (ROS) and reducing oxidative damage in various tissues. *Euglena* paramylon, and its aqueous extract constructed with chitosan-hyaluronic acid hydrogel facilitate cutaneous wound healing in mice without inducing excessive inflammatory response (Li et al., 2021). Animal studies have highlighted its role in protecting liver and kidney tissues from oxidative stress, as well as enhancing wound healing through angiogenesis and collagen deposition (Yasuda et al., 2018; Xie et al., 2021).

In functional foods and nutraceuticals, paramylon has been successfully incorporated into products aimed at weight management and cholesterol reduction. Its unique structural properties and bioactivity make it an ideal ingredient for developing next-generation functional foods. Moreover, its safety and efficacy in enhancing immune responses have opened avenues for its use in animal feed, particularly in aquaculture and livestock. Supplementation with paramylon in fish diets has been shown to improve growth performance and reduce susceptibility to infections (Bashir and Choi, 2017; Yamamoto et al., 2018; Hayatgheib et al., 2020).

### 2.4 Challenges and future directions

Despite its promising potential, several challenges remain in the large-scale cultivation of *Euglena* and the optimization of paramylon extraction. Cost-effective and sustainable cultivation systems, such as advanced photobioreactors and nutrient recycling strategies, are essential to increase production efficiency (Fujita et al., 2008; Vogler et al., 2018). Additionally, exploring chemical modifications of paramylon to enhance its solubility and bioavailability could further expand its applications (Feuzing et al., 2022; Li et al., 2024).

Future research should prioritize large-scale clinical trials to validate the health benefits observed in preclinical studies. Such trials would provide robust evidence for its efficacy and broaden its acceptance in global markets (Ullmann and Grimm, 2021; Wang Q. et al., 2024). Moreover, interdisciplinary collaborations combining synthetic biology, metabolic engineering, and material sciences could unlock novel applications for paramylon, from drug delivery systems to bioactive packaging materials (Monfils et al., 2011; He L. et al., 2024).

### 2.5 Conclusion

*Euglena*-derived β-glucan, or paramylon, is a remarkable biomolecule with significant potential across diverse industries. Its high yield, ease of extraction, and versatile bioactivities make it a superior alternative to traditional β-glucan sources. By addressing current challenges and exploring innovative applications, paramylon can play a pivotal role in advancing functional foods, pharmaceuticals, and sustainable animal health solutions.

## 3 Microalgae in drug delivery systems

Microalgae are emerging as innovative platforms in drug delivery systems due to their unique biological properties, including biocompatibility, natural abundance, and the ability to be genetically modified. Their cellular structures, bioactive compounds, and sustainable cultivation methods make them ideal candidates for developing advanced drug delivery technologies (Geng et al., 2024; Rakhi et al., 2025).

### 3.1 Advantages of microalgae in drug delivery

Microalgae offer several key advantages as carriers in drug delivery systems. Firstly, their cell walls are naturally composed of biocompatible polysaccharides such as cellulose and chitin, which can be tailored to encapsulate and release pharmaceutical compounds in a controlled manner (Geng et al., 2024; Rakhi et al., 2025). Additionally, microalgae-derived lipids and proteins can be engineered to form nanoparticles or vesicles, enhancing the bioavailability of hydrophobic drugs (Dubey et al., 2023).

Moreover, microalgae possess intrinsic bioactivities, including antioxidant and anti-inflammatory properties, which can synergize with therapeutic agents. For example, carotenoids like astaxanthin and lutein have been shown to protect drug molecules from oxidative degradation, improving their stability and efficacy (Aslam et al., 2021; Pereira et al., 2024). Furthermore, their natural ability to accumulate bioactive compounds allows for simultaneous production of therapeutic agents and drug carriers, reducing manufacturing complexity (Lu et al., 2024).

### 3.2 Applications in targeted drug delivery

One of the most promising applications of microalgae in drug delivery is their role in targeted therapies. Genetically engineered microalgae can express surface ligands that bind specifically to cancer cells or inflamed tissues. For instance, *C*. *reinhardtii* has been modified to deliver monoclonal antibodies to tumor sites, demonstrating high specificity and minimal off-target effects (Delalat et al., 2015; Dubey et al., 2023).

Another innovative approach involves the use of microalgae-derived extracellular vesicles (EVs) for drug delivery. These vesicles, secreted naturally by microalgae, can be loaded with small-molecule drugs, nucleic acids, or proteins, and have been shown to cross biological barriers such as the blood-brain barrier (Adamo et al., 2021; Adamo et al., 2024).

### 3.3 Microalgae-based oral delivery systems

Microalgae have also been explored for oral drug delivery due to their resistance to gastrointestinal conditions. Encapsulation of probiotics and bioactive compounds in algal cells such as *Spirulina platensis* protects these agents from acidic and enzymatic degradation, ensuring their release in the intestinal tract (Zhong et al., 2021; Zhang et al., 2022). Additionally, algal-derived polysaccharides like alginate and carrageenan are widely used as matrices for oral drug formulations due to their excellent mucoadhesive and gel-forming properties (Geng et al., 2024). An oral microsphere strategy (Eug/Lut@HAMA) was developed by encapsulating *Euglena* (Eug) and luteolin (Lut) within methacrylated hyaluronic acid (HAMA) microspheres as a potential treatment for hyperuricemia with renal injury (Liu et al., 2025).

### 3.4 Challenges in scaling microalgae-based drug delivery

Microalgae-based drug delivery systems, despite their immense potential, face several challenges that must be overcome for widespread application. Firstly, the standardization of cultivation and processing is crucial due to the variability in algal biomass composition caused by environmental and cultivation conditions, which affects the consistency of drug carrier production. To address this, the development of standardized protocols and closed bioreactor systems can help mitigate these issues (Sharma et al., 2024; Kapoor et al., 2024). Secondly, the efficiency of drug loading and release within algal systems needs optimization, with encapsulation efficiency and controlled release kinetics requiring attention. Advances in bioengineering and nanotechnology could be instrumental in improving these parameters (Patra et al., 2018; Nallasamy and Natarajan, 2021). Thirdly, regulatory hurdles present a significant barrier since microalgae-based carriers are a relatively new concept, and regulatory frameworks for their approval in pharmaceutical applications are underdeveloped. Comprehensive safety assessments and clear guidelines are essential to facilitate market entry (Su et al., 2023). Lastly, the cost-effectiveness of scaling up microalgae cultivation and processing for pharmaceutical-grade applications is a concern, as it can be expensive. However, integrating drug delivery with other algal-based industries, such as biofuels and nutraceuticals, may help offset production costs (Grama et al., 2022).

### 3.5 Future directions and innovations

The integration of synthetic biology with microalgae research holds significant promise for drug delivery. Advances in genome editing tools such as CRISPR-Cas9 enable precise engineering of algal cells to enhance drug loading capacities and targeting efficiencies (Patel et al., 2023; Ambily et al., 2024). Additionally, the development of hybrid systems combining microalgae with synthetic nanocarriers, such as liposomes or dendrimers, could further expand their applications in complex therapeutic regimens (Huang et al., 2024).

Emerging technologies such as microfluidics and AI-driven design are expected to accelerate the development of microalgae-based drug delivery systems. These tools can optimize algal cultivation, drug encapsulation, and release profiles, ensuring scalability and clinical efficacy (Liang et al., 2015; Ummalyma et al., 2020). Furthermore, interdisciplinary collaborations among biologists, engineers, and pharmaceutical scientists will be essential to translate these innovations into commercial products.

## 4 Synthetic biology applications in microalgae

Synthetic biology integrates biology and engineering to design, construct, and modify biological systems for practical applications. Microalgae, as versatile photosynthetic organisms, provide a promising platform for synthetic biology due to their ability to produce a diverse range of bioactive compounds, high biomass yields, and potential environmental benefits. With the rapid advancement of genome editing tools and metabolic engineering techniques, synthetic biology in microalgae has entered a transformative phase (Hu et al., 2023; Jeong et al., 2023).

### 4.1 Synthetic biology tools and genetic engineering

Modern synthetic biology in microalgae is driven by genome-editing tools like CRISPR-Cas9, TALENs, and ZFNs. For example, CRISPR-Cas9 was successfully applied in *C*. *reinhardtii* to knock out genes involved in starch synthesis, which significantly increased lipid production (Baek et al., 2016). Using TALENs technique, the gene encoding the urease enzyme in the model diatom, *Phaeodactylum tricornutum*, was targeted for interruption (Weyman et al., 2015). Similarly, TALENs have been employed in *Nannochloropsis* to enhance the expression of key genes in the fatty acid biosynthesis pathway, boosting biodiesel precursor yields (Song et al., 2024). These genome-editing tools, combined with universal cloning systems, allow researchers to precisely modify genetic pathways and introduce new functionalities (Patwari et al., 2023). A notable case involved engineering *Haematococcus pluvialis* to produce higher concentrations of astaxanthin, a valuable antioxidant with applications in nutraceuticals (Gu et al., 2024). High-throughput sequencing revealed low-efficacy genome editing using Cas9 RNPs electroporation and single-celled microinjection provided an alternative to deliver CRISPR reagents into *E*. *gracilis* (Chen et al., 2022a) and a synthetic biology perspective on the bioengineering tools for an industrial biotechnology platform were being built up recently (Chen et al., 2022b).

### 4.2 Applications in metabolic engineering

Synthetic biology has enabled the enhancement of metabolic pathways in microalgae to produce valuable compounds. In the case of fatty acids production, the engineered *N. oceanica* produced more eicosapentaenoic acid (EPA), a high-value ω-3 polyunsaturated fatty acids, than wild type (Liu et al., 2024). Another study applied CRISPR-Cas9 to improve the triacylglycerol pathway in *Dunaliella salina*, leading to higher biodiesel yields (Hu et al., 2023). Pigments and nutraceuticals have also benefited, with engineered strains producing increased levels of carotenoids and phycocyanin (Cao et al., 2023). For instance, *P. tricornutum* was engineered to produce fucoxanthin, a carotenoid with anti-cancer and anti-inflammatory properties, demonstrating the scalability of such innovations (Wu S. et al., 2023).

### 4.3 Advances in photosynthetic efficiency and carbon fixation

Improving photosynthetic efficiency and carbon fixation pathways has been a major focus of synthetic biology in microalgae. One approach involves reducing antenna sizes in photosystems to minimize energy loss via non-photochemical quenching (NPQ). For example, modifications in *Nannochloropsis gaditana* led to a 20% increase in photosynthetic efficiency (Negi et al., 2020). Additionally, engineering the Calvin-Benson-Bassham cycle in *C*. *reinhardtii* by incorporating synthetic carbon fixation pathways significantly improved carbon assimilation rates (Boisset et al., 2023). Another approach involved incorporating cyanobacterial carbon-concentrating mechanisms into *Chlorella vulgaris*, enhancing its ability to fix CO_2_ under high-light conditions (Long et al., 2016).

### 4.4 Biosensors: enhancing efficiency in microalgal systems

Biosensors have emerged as powerful tools in synthetic biology, enabling real-time monitoring of cellular and environmental parameters. These genetically encoded sensors can detect specific metabolites or environmental factors, generating measurable signals such as fluorescence or luminescence. Real-time metabolite monitoring has been achieved in genetically engineered *C*. *vulgaris*, where fluorescent biosensors tracked lipid accumulation, allowing for dynamic adjustments in cultivation conditions (Antonacci and Scognamiglio, 2020; Patwari et al., 2023).

Moreover, biosensors have been employed to optimize nitrate and phosphorus utilization in photobioreactors. For instance, nitrate-responsive biosensors in *Synechococcus elongatus* provided real-time data on nutrient availability, enabling precise adjustments to nutrient supply and improving nitrogen use efficiency (Hasan et al., 2024). Another innovation involved the use of biosensors for detecting reactive oxygen species (ROS), which helped monitor oxidative stress in *C*. *reinhardtii*, ensuring optimal cultivation conditions (Molen et al., 2006).

High-throughput screening enabled by biosensors has accelerated the identification of high-performing genetic variants. For example, a biosensor-assisted selection system in *P*. *tricornutum* identified strains with enhanced fucoxanthin production, achieving a 40% increase in yield (Macdonald Miller et al., 2023). Additionally, biosensors integrated with wireless systems are being explored for remote monitoring in large-scale outdoor photobioreactors, demonstrating their potential to scale microalgal cultivation (Heining et al., 2015).

### 4.5 Emerging biosensor technologies

Emerging biosensor technologies are revolutionizing the field of synthetic biology in microalgae by introducing advanced detection capabilities and integration with automated systems. One notable advancement is the development of multi-analyte biosensors capable of simultaneously detecting various metabolites, such as glucose, nitrate, and lipid precursors, within a single cultivation system. These biosensors leverage nanoscale materials, including graphene and gold nanoparticles, to achieve high sensitivity and specificity (Huang et al., 2021; Parkhe and Tiwari, 2024).

The application of microfluidic biosensors is another frontier. These devices integrate biosensing components into miniaturized platforms, enabling real-time monitoring of microalgae cultures with minimal sample requirements. For instance, microfluidic chips embedded with biosensors have been used to monitor pH and nutrient gradients in dynamic cultivation environments, providing unparalleled insights into cellular responses under stress conditions (Pattanayak et al., 2021).

Additionally, wearable biosensors have been developed for real-time monitoring of outdoor photobioreactor systems. These devices are equipped with wireless data transmission capabilities, allowing operators to remotely monitor critical parameters such as oxygen evolution and light absorption efficiency. Such technologies are particularly valuable for large-scale operations where manual monitoring is impractical (Xu et al., 2024).

Another promising innovation involves the integration of biosensors with CRISPR-based detection systems. These CRISPR-biosensors exploit the sequence-specific detection capabilities of CRISPR-Cas systems to identify genetic markers indicative of desired traits or stress responses. For example, CRISPR-biosensors have been employed to detect the activation of lipid biosynthesis pathways in *Nannochloropsis* cultures, enhancing the precision of metabolic engineering efforts (Xie et al., 2024).

These emerging technologies are poised to significantly enhance the scalability, efficiency, and precision of microalgae cultivation. By integrating these biosensors into synthetic biology workflows, researchers can achieve more robust control over metabolic pathways, optimize cultivation conditions, and expand the industrial applicability of microalgae-derived products.

### 4.6 Challenges and future directions

Despite these advancements, several challenges remain in scaling synthetic biology applications for microalgae. Photobioreactor design and operation present a major hurdle, as scaling up these systems requires maintaining consistent light exposure, temperature, and nutrient distribution. Current large-scale systems often struggle with suboptimal light penetration and uneven CO_2_ distribution, which reduces overall productivity. To overcome these issues, advancements in photobioreactor design, such as modular systems with adaptive lighting technologies, are necessary (Huang et al., 2017; Patwari et al., 2023). Additionally, high production costs associated with genetic engineering, cultivation, and downstream processing challenge the economic feasibility of synthetic biology applications. While techniques like CRISPR-Cas9 are effective, they remain costly for industrial-scale deployment, making the development of cost-effective genome-editing tools and high-throughput screening methods crucial (Sarkar et al., 2023).

The genomic complexity and resource limitations of many microalgae species also hinder the optimization of metabolic pathways. Limited genomic resources, including incomplete genome annotations and poorly understood regulatory networks, are a significant barrier that can be overcome with improved genomic databases and functional annotation pipelines (Stavridou et al., 2024). Regulatory and biosafety concerns arise when deploying genetically modified microalgae in open environments for biofuel or environmental applications. Developing stricter international guidelines and risk assessment protocols is essential to ensure safe and sustainable use (Henley et al., 2013).

The scalability of cultivation systems is another challenge, as the transition from laboratory to industrial-scale cultivation introduces variability in growth rates, biomass yields, and metabolic performance. Advanced photobioreactor designs and innovative cultivation strategies are needed to ensure consistent performance across different scales (Dharmaraja et al., 2023). Lastly, public acceptance and market integration are critical, as consumer skepticism regarding genetically modified organisms (GMOs) may slow the adoption of synthetic biology-derived products. Public awareness campaigns that highlight the environmental and economic benefits of these innovations will be crucial to gaining market acceptance (Hwang and Nam, 2021).

Addressing these challenges requires interdisciplinary collaborations among biologists, engineers, and computational scientists. Future innovations will likely focus on more efficient genetic tools, enhanced metabolic engineering techniques, and robust systems for scaling production.

## 5 Challenges and solutions

Microalgae-based innovations, despite their immense potential, face a range of challenges across technical, economic, consumer acceptance and regulatory dimensions that must be addressed to achieve large-scale adoption and practical application. Fortunately, solutions to these barriers are emerging, paving the way for scalable and sustainable applications.

### 5.1 Technical challenges and solutions

The technical challenges and solutions in microalgae-based innovations are multifaceted. Cultivation constraints are a significant hurdle, as large-scale cultivation of microalgae is complex. Open-pond systems, while cost-effective, are prone to contamination and environmental variability. In contrast, closed photobioreactors offer better control but come with significant capital and operational costs (Jerney and Spilling, 2020; Novoveská et al., 2023). Solutions to these constraints include the development of advanced modular photobioreactors with adaptive lighting systems, which optimize light distribution and reduce energy consumption. Additionally, integrating CO_2_ capture technologies with cultivation systems can enhance resource utilization efficiency (Gupta et al., 2015; Skifa et al., 2025). Genetic stability is another challenge, as genetic modifications in engineered microalgae often face stability issues across multiple cultivation cycles. To overcome this, researchers are focusing on stable genome editing techniques, such as base editing and epigenetic modifications, to minimize off-target effects while preserving productivity. Advanced bioinformatics tools are also being used to predict and mitigate genetic instability (Jeong et al., 2023; Onn et al., 2024). Lastly, in the context of drug delivery systems, improving the encapsulation efficiency and controlled release profiles of microalgae-based carriers is technically demanding. Nanotechnology-based drug encapsulation and controlled-release systems, coupled with machine learning algorithms for optimizing release kinetics, are being developed to enhance drug delivery efficiency (Kang et al., 2024; Wang X. et al., 2024).

### 5.2 Economic challenges and solutions

High production costs are a significant hurdle, especially for microalgae-derived products like β-glucans and engineered therapeutic compounds. Efforts to address these challenges include the integration of co-products, such as biofuels and animal feed, to improve overall economic viability. Additionally, leveraging public-private partnerships can provide funding and reduce the economic risks associated with large-scale production (Wang X. et al., 2024; Skifa et al., 2025). Market integration is another challenge. Competing with established agricultural and chemical synthesis methods remains challenging. Developing strong marketing strategies highlighting the environmental benefits and health advantages of microalgae products can help penetrate competitive markets. Certification schemes and eco-labels emphasizing sustainability are being explored to attract eco-conscious consumers (Ramadas et al., 2021; Zhong et al., 2023).

### 5.3 Consumer acceptance challenges and solutions

The commercialization of microalgae in healthcare and functional foods has gained momentum due to their nutritional benefits and potential to address global health challenges. However, consumer acceptance remains a critical factor in determining their market success. Key elements influencing consumer acceptance include taste, cost-effectiveness, and public perception, each of which plays a pivotal role in shaping purchasing decisions and long-term adoption.

Taste is one of the most significant barriers to consumer acceptance of microalgae-based products. Microalgae, such as *Spirulina* and *Chlorella*, often have distinct flavors that can be perceived as unpleasant, such as earthy, fishy, or bitter notes (Becker, 2007). These sensory characteristics can deter consumers. To overcome this challenge, manufacturers are increasingly investing in flavor-masking technologies and incorporating microalgae into familiar food formats, such as snacks, beverages, and baked goods. For example, *Spirulina* has been successfully integrated into smoothies and energy bars, where its flavor is less pronounced (Caporgno and Mathys, 2018). Cost-effectiveness is another critical factor influencing consumer acceptance. Microalgae cultivation and processing can be expensive due to the need for controlled environments, specialized equipment, and high-quality inputs. These costs are often passed on to consumers, resulting in higher retail prices compared to conventional functional foods (Çelekli et al., 2024). To address this, advancements in cultivation technologies, such as photobioreactors and waste-based nutrient sources, are being explored to reduce production costs and improve affordability (Taparia et al., 2016). Public perception of microalgae-based products also plays a crucial role in their commercial success. Despite their nutritional benefits, microalgae are often perceived as unconventional or unappealing, particularly in Western markets where they are not traditionally consumed (Plaza et al., 2008). Misconceptions about safety, sustainability, and efficacy can further hinder consumer acceptance. Effective communication strategies, including clear labeling, educational campaigns, and endorsements from trusted health authorities, are essential to build trust and awareness. For instance, highlighting the environmental sustainability of microalgae cultivation, such as their low carbon footprint and ability to grow in non-arable land, can appeal to environmentally conscious consumers (Borowitzka, 2013).

### 5.4 Regulatory challenges and solutions

The utilization of microalgae in therapeutics and functional foods has gained significant attention due to their rich nutritional profiles and potential health benefits. However, the regulatory landscape for these products varies considerably across different regions, posing unique challenges for market entry and compliance. In the United States, microalgae-based products are regulated by the Food and Drug Administration (FDA) under the categories of dietary supplements, food additives, or drugs, depending on their intended use. For instance, *Spirulina* and *Chlorella* are generally recognized as safe for use in foods, but any health claims must be substantiated by rigorous clinical trials (Hosny et al., 2025). The FDA’s stringent requirements for safety and efficacy data can be a significant hurdle, particularly for novel microalgae strains or extracts. In the European Union (EU), microalgae products fall under the jurisdiction of the European Food Safety Authority (EFSA). Health claims related to microalgae must be approved under the EU Health Claims Regulation, which demands robust scientific evidence, often involving human intervention studies (Bigliardi and Galati, 2013). This process can be time-consuming and costly, particularly for small and medium-sized enterprises. In Asia, particularly in countries like China and Japan, the regulatory environment is somewhat more accommodating but still complex (Patel et al., 2008). The approval process for new functional foods can be lengthy, requiring extensive documentation and testing. Japan, on the other hand, has a well-established system for functional foods under the Foods with Function Claims (FFC) system, which allows for a more streamlined approval process. However, the specific requirements for microalgae-derived products can still be challenging to navigate, particularly regarding the substantiation of health benefits. In developing regions, such as parts of Africa and South America, the regulatory frameworks for microalgae-based products are often less developed. This can lead to inconsistencies in enforcement and a lack of clarity for manufacturers. However, the absence of stringent regulations can also provide opportunities for rapid market entry, albeit with potential risks related to product quality and safety (Salehipour-Bavarsad et al., 2024).

Regulatory challenges in the microalgae biotechnology sector require thoughtful solutions. The approval processes for microalgae-based products are still evolving. Harmonizing international regulatory standards and creating streamlined safety assessment protocols for genetically modified strains can reduce barriers to market entry. Collaborations between regulatory bodies and researchers are vital to accelerate approval processes (Herrmann et al., 2021; Onn et al., 2024). Additionally, public perception plays a significant role, as consumer skepticism toward genetically modified organisms (GMOs) can hinder acceptance. Public outreach campaigns and transparent labeling practices are being implemented to improve consumer trust. Demonstrating clear safety profiles through large-scale clinical studies is also key to alleviating skepticism (Ramadas et al., 2021; Kang et al., 2024). By addressing these challenges through technological advancements, economic strategies, and regulatory alignment, the field of microalgae biotechnology can unlock its full potential for transformative impact.

## 6 Perspectives

The integration of microalgae into biotechnology represents a transformative opportunity to address global challenges in health, nutrition, and sustainability. Recent advancements in microalgae research have highlighted three critical areas for future development: technological innovations, sustainability approaches, and collaborative networks. These interconnected strategies are key to overcoming current limitations and unlocking the full potential of microalgae-based applications.

### 6.1 Technological innovations

Technological advancements are reshaping the scalability and precision of microalgae cultivation. The development of advanced photobioreactors, equipped with adaptive lighting systems and modular designs, has significantly improved light utilization efficiency, reducing energy consumption and operational costs. For instance, modular photobioreactors used in large-scale *C*. *vulgaris* production demonstrated a 30% increase in biomass yield under controlled light conditions compared to conventional systems (Penloglou et al., 2024). Moreover, genome-editing tools, such as CRISPR-Cas9 and TALENs, are enabling precise metabolic modifications in microalgae (Jeon et al., 2017)​. A recent study on *N*. *oceanica* reported a 40% enhancement in lipid productivity through targeted pathway optimization (Liu et al., 2024)​. These technologies not only enhance production efficiency but also broaden the spectrum of valuable compounds derived from microalgae, including biofuels, nutraceuticals, and therapeutic proteins.

### 6.2 Sustainability approaches

Integrating microalgae cultivation with environmental sustainability initiatives offers dual benefits: economic feasibility and ecological impact. Microalgae-based carbon capture systems are being developed to mitigate industrial CO_2_ emissions while simultaneously promoting biomass growth. For example, a pilot project in Germany combined microalgae bioreactors with cement factory emissions, achieving a 50% reduction in carbon output while producing valuable algal biomass for biofuel production (He Z. et al., 2024). Additionally, using microalgae for wastewater treatment has proven effective in recovering nutrients and reducing contaminants. *S*. *obliquus* cultivation in municipal wastewater not only reduced nitrogen and phosphorus levels by over 80% but also generated algal biomass rich in proteins and lipids​ (de Morais et al., 2023). These integrated approaches underscore the economic and environmental potential of microalgae as a sustainable resource.

### 6.3 Collaborative networks

The rapid adoption and innovation in microalgae biotechnology depend on strong partnerships between academia, industry, and regulatory bodies. Collaborative networks facilitate knowledge exchange, resource sharing, and streamlined commercialization pathways. For instance, public-private partnerships in Asia have accelerated the development of genetically modified microalgae for aquaculture feed, addressing regional food security challenges (Ahmad and Ashraf, 2024). Establishing international regulatory standards through such collaborations will further reduce barriers to market entry and promote global acceptance of microalgae-based innovations.

## 7 Conclusion

Microalgae-based innovations in β-glucan production, drug delivery systems and synthetic biology applications hold transformative potential. These advancements not only promise revolutionary changes in health, nutrition, and environmental sustainability but also set a precedent for biotechnological solutions to global challenges.

The utilization of β-glucans underscores microalgae’s inherent bioactivity, with applications ranging from immune enhancement to metabolic health. These natural compounds, particularly paramylon from *Euglena*, showcase the potential to bridge functional foods and therapeutic innovations. Synthetic biology has further amplified microalgae’s role in biotechnology by providing precise tools to enhance productivity and expand applications. Tools like CRISPR-Cas9 and TALENs have opened avenues for producing high-value compounds such as biofuels, pigments, and pharmaceuticals, while reducing costs and improving scalability.

Environmental sustainability remains a cornerstone of microalgae’s appeal. Integrating carbon capture and wastewater treatment with microalgae cultivation demonstrates dual benefits: reducing environmental pollutants and generating valuable biomass. Successful case studies, such as nutrient recovery in wastewater or CO_2_ mitigation in industrial emissions, exemplify microalgae’s potential for circular economy initiatives.

Finally, collaborative networks between academia, industry, and regulatory bodies are crucial. These partnerships accelerate innovation, streamline commercialization, and enhance consumer trust through transparent communication. For instance, international consortia have developed cost-efficient methods for microalgal production, making it feasible for widespread applications in nutraceuticals and beyond.

By addressing technical, economic, and regulatory challenges through targeted solutions, microalgae biotechnology stands at the forefront of sustainable and impactful innovation. This comprehensive integration of bioactive research, engineering, and practical applications positions microalgae as a critical contributor to future global sustainability and health advancements.
